# Metacaspase-binding peptide inhibits heat shock-induced death in *Leishmania (L.) amazonensis*

**DOI:** 10.1038/cddis.2017.59

**Published:** 2017-03-02

**Authors:** Mauricio S Peña, Guilherme C Cabral, Wesley L Fotoran, Katia R Perez, Beatriz S Stolf

**Affiliations:** 1 Department of Parasitology, Institute of Biomedical Sciences, University of Sao Paulo, Sao Paulo, Brazil; 2 Department of Biophysics, Federal University of Sao Paulo, Sao Paulo, Brazil

## Abstract

*Leishmania (Leishmania) amazonensis* is an important agent of cutaneous leishmaniasis in Brazil. This parasite faces cell death in some situations during transmission to the vertebrate host, and this process seems to be dependent on the activity of metacaspase (MCA), an enzyme bearing trypsin-like activity present in protozoans, plants and fungi. In fact, the association between MCA expression and cell death induced by different stimuli has been demonstrated for several *Leishmania* species. Regulators and natural substrates of MCA are poorly known. To fulfill this gap, we have employed phage display over recombinant *L. (L.) amazonensis* MCA to identify peptides that could interact with the enzyme and modulate its activity. Four peptides were selected for their capacity to specifically bind to MCA and interfere with its activity. One of these peptides, similar to ecotin-like ISP3 of *L. (L.) major*, decreases trypsin-like activity of promastigotes under heat shock, and significantly decreases parasite heat shock-induced death. These findings indicate that peptide ligands identified by phage display affect trypsin-like activity and parasite death, and that an endogenous peptidase inhibitor is a possible natural regulator of the enzyme.

*Leishmania (Leishmania) amazonensis* is the second most frequent agent of cutaneous leishmaniasis in Brazil,^1^ a country with high incidence of this disease (WHO 2016).

The promastigote forms of *Leishmania* are transmitted to man and other mammals by the bite of an infected female sand fly. Once inside vertebrate phagocytes, promastigotes convert into the amastigote forms, responsible for disease progression.^2, 3^ Macrophages are the parasite's main host cells that nevertheless possess several mechanisms to restrain the infection such as by nitric oxide (NO) and reactive oxygen species (ROS) production.^4, 5^

After transmission to the vertebrate host, promastigotes face a heat shock and an oxidative attack of the innate immune system. The exposure of promastigotes to NO, ROS, hydrogen peroxide,^6^ heat shock^7, 8^ and drugs^9, 10, 11^ induces phenotypical changes characteristic of programmed cell death (PCD) such as cell shrinkage, DNA fragmentation, activation of peptidases^12^ and exposition of a ‘PS-like' (annexin V binding) phospholipid.^13, 14^

PCD by apoptosis is considered to be dependent on the activation of caspases, cysteine-dependent peptidases.^15^ Plants, fungi and protozoa do not code for caspases but express metacaspases (MCAs).^16^ MCAs are cysteine peptidases from clan CD, family C14, that share the histidine-cysteine catalytic dyad with caspases, but differently from caspases, which are specific to aspartic acid at P1, are specific to arginine/lysine, that is, have trypsin-like activity.^16, 17, 18, 19^ MCAs have been shown to have caspase-like auto-processing,^20^ and for some *Leishmania* species,^20, 21^ as well as yeast ^22^ and *Arabidopsis*,^23, 24^ processing of the enzyme is essential for activity.

*Leishmania* MCA was first described in *Leishmania (L.) major*^20^ and in *Leishmania* (*L.) donovani*,^25^ and was later identified in *Leishmania (L.) mexicana*^26^ and *Leishmania (L.) infantum*.^9^ Most *Leishmania* species have a single MCA gene, while *L. (L.) infantum* and *L. (L.) donovani* have two genes that code for proteins with 96% identity.^27^ MCA has important roles not only in cell death but also in the control of amastigote intracellular proliferation^26^ and autophagy.^26, 28^

The association between MCA and cell death has first demonstrated in yeast^22^ and *Trypanosomabrucei*,^29^ and later in *Arabidopsis*^30^ and different *Leishmania* species. Oxidative stress caused MCA-dependent cell death in *Saccharomyces cerevisiae*, and the yeast knock out strain was efficiently complemented by *Leishmania (L.) major* MCA.^20^ Accordingly, oxidative stress induced higher ‘PS-like' exposure in a *Leishmania (L.) major* lineage that over expressed MCA catalytic region.^21^ Drugs such as miltefosine induced death in *Leishmania (L.) infantum* associated with MCA overexpression,^9^ and a *Leishmania (L.) major* MCA-deficient lineage was resistant to miltefosine and curcumin.^28^

Regulators and natural substrates of MCA were not frequently studied. It was recently shown by two-hybrid system that *Leishmania (L.) major* mitogen-activated protein kinase MPK7 and calpain interact with the C-terminal domain of MCA, probably participating on the induction of parasite death.^28^ No similar study was ever performed on *Leishmania (L.) amazonensis* MCA or other *Leishmania* species.

Phage display is an effective tool for searching for protein ligands, allowing the identification of natural protein interactions and of potential synthetic modulators.^31, 32^ In this work we have employed a commercial phage display library over recombinant *L. (L.) amazonensis* MCA to identify peptides that could interact with the enzyme, modulating its activity and eventually affecting parasite survival. We identified peptides that specifically bind to MCA. One of them decreases trypsin-like activity in promastigotes under heat shock and reduces parasite heat shock-induced death. This peptide is similar to the *L. (L.) major* ecotin-like ISP3,^33^ a potent inhibitor of several serine proteases.^34^

Our study is the first to show that MCA can be inhibited by a peptide similar to a parasite-coded serine protease inhibitor. Besides, we show that phage display can be effectively used to identify modulators of specific *Leishmania* targets, suggesting that this technique can be employed to decipher poorly known processes and to search for potential parasite-specific drugs.

## Results

### Heat shock induces *L. (L.) amazonensis* promastigote death and increases trypsin-like activity

**Figure 1:**
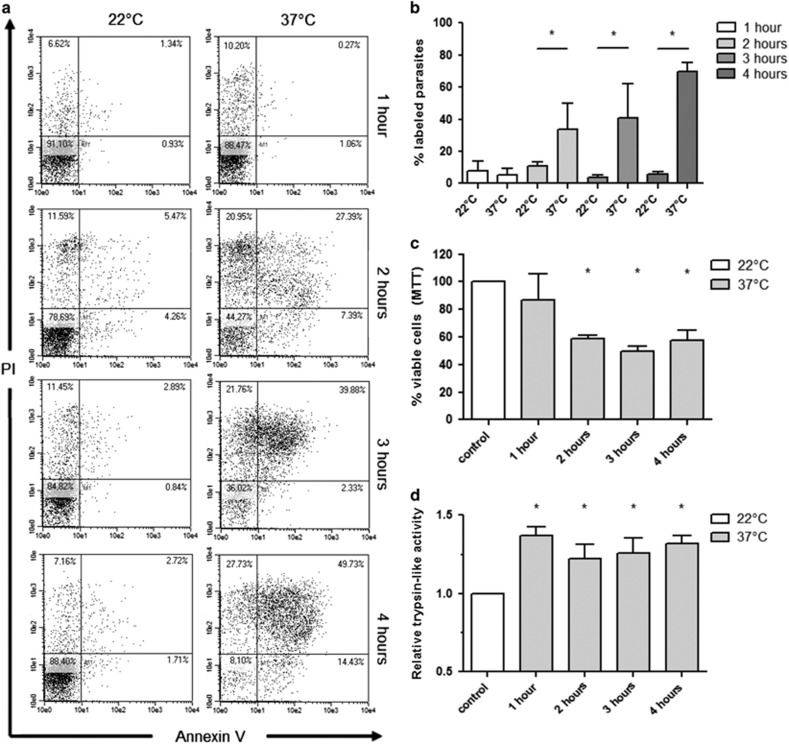
Heat shock induces death of *L. (L.) amazonensis* promastigotes and increases trypsin-like activity. (**a**) Flow cytometry plots for annexin V and PI labeling of promastigotes incubated at 22 or 37 °C for 1, 2, 3 or 4 h. (**b**) Percentage of parasites with annexin V or annexin V+PI labeling (named as % labeled parasites) by flow cytometry (controls in Supplementary Figure 1). (**c**). MTT assay showing viability after 1, 2, 3 and 4 h of heat shock (37 °C), each of them relative to the incubation at 22 °C for the same period, considered as 100%. (**d**). Trypsin-like activity (named as relative trypsin-like activity) using Z-Arg-Arg-AMC substrate and 2 *μ*g of extract of promastigotes incubated at 37 °C, relative to the extract obtained after incubation at 22 °C for the same period, considered as 1.0. For all figures, data represent means and standard deviations of three independent experiments. Statistical analysis by ANOVA followed by Tukey, *P<0.05

*Leishmania* faces heat shock during transmission to the vertebrate host, and this event induces death of a part of the parasite's population.^8^ We analyzed the effect of heat shock at 37 °C (compared with 22 °C, the sand fly temperature) for different periods in promastigote death employing MTT assay and annexin V and PI labeling. The results shown in Figures 1(a–c) indicate that viability is significantly diminished after 2 (based on MTT, c) and 3 h (based on annexin V and PI, a, b) of heat shock.

MCA has already been suggested to be involved in *Leishmania* heat shock-induced cell death.^21^ We thus quantified trypsin-like activity in parasite extracts after incubation at 37 °C. Figure 1c demonstrates that heat shock for 1, 2, 3 and 4 h significantly increased activity, reinforcing the possible role for the enzyme in this process.

### Production of active recombinant *L. (L.) amazonensis* MCA

**Figure 2:**
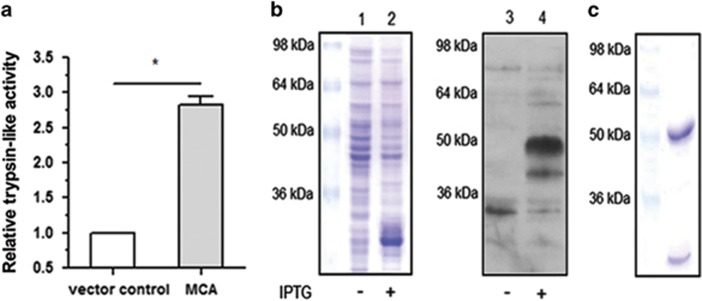
Recombinant MCA was effectively produced in bacterial system. (**a**) Trypsin-like activity (named as relative trypsin-like activity) measured in 20 *μ*g of bacterial extract transformed with control vector (pET-28a) or MCA-containing plasmid, induced with IPTG. Results of three experiments with technical triplicates. Statistical analysis by *t*-test, *P<0.05. (**b**) SDS-PAGE (lanes 1 and 2) and western blot (lanes 3 and 4) using anti-his for detection of MCA in bacterial system with (+) or without (−) IPTG induction. (**c**) SDS-PAGE showing recombinant MCA after purification

Little is known about MCA substrates and regulators. Aiming to find natural or artificial ligands that could modulate enzyme activity and eventually parasite death, we employed the phage display technique on the recombinant enzyme. Bacteria were transformed with pET-28a vector containing *L. (L.) amazonensis* MCA gene and trypsin-like activity was assayed in extracts (Figure 2a). Activity was higher in bacteria transformed with MCA containing plasmid, as already described for plant MCA in the same system.^24^ This result indicated that bacteria could produce a functional (active) recombinant protein. His-tagged protein with approximately 50 kDa was identified in bacterial extracts after induction with IPTG (Figure 2b), and was purified along with a processed form of ~25 kDa (Figure 2c), already described.^23^

### Identification of MCA-binding peptides able to modulate enzyme activity

Panning using the commercial Ph.D.-7 Phage Library (phages containing seven random amino acids in pIII protein) was performed over the immobilized MCA protein. Three cycles were performed, and a small enrichment in the number of bound phages was observed after each cycle (Supplementary Table I). After three cycles, 50 bound phages were randomly picked and sequenced. The sequences of the corresponding peptides and their frequencies are shown in Table 1.

**Figure 3:**
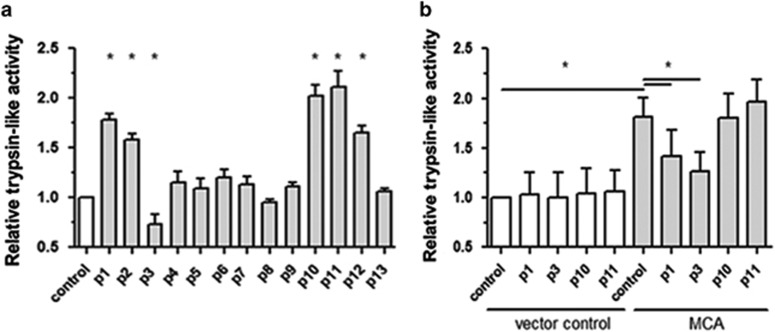
Peptides modulate trypsin-like activity in promastigotes and in bacteria expressing MCA. Relative trypsin-like activity of (**a**). Promastigote extracts pre-incubated with each of the 13 selected peptides (100 *μ*M) or with DMSO (control). (**b**) Bacteria expressing MCA or transformed with control vector (pET-28a), pre-incubated with p1, p3, p10 and p11 peptides (100 *μ*M) or with DMSO (control). Results of three experiments with technical triplicates, normalized by the corresponding activity in DMSO 0,5% (represented as 1.0). Statistical analysis by ANOVA followed by Tukey. *P<0.05 relative to control

Phage display selection usually leads to a large number of different bound phages, and selection of the ones to be validated is usually based on their frequencies.^35, 36, 37^ Thirty-three different peptides were encoded by the 50 phages sequenced. Most of them were observed in only one phage, seven were present in two phages, one in four phages and two in five phages (see Table 1). We thus selected 13 peptides (named 1–13, shown in Table 1) using as criteria the identification of the peptide in more than one phage or the presence of a three amino acid repeat shared with another peptide. These peptides were synthesized and the effect of each of them on trypsin-like activity in promastigote extracts and in extracts of bacteria transformed with control vector or MCA plasmid is shown in Figure 3.

Figure 3a shows that peptides 1, 2, 10, 11 and 12 significantly increased and peptide 3 significantly decreased trypsin-like activity in promastigotes compared with control. To prove that the peptides' effects on the promastigote trypsin-like activity were due to their interaction with MCA and not with other enzymes, we analyzed the effects of peptides 1, 3, 10 and 11 on trypsin-like activity in bacteria extracts. Trypsin-like activity of bacteria expressing or not MCA in the presence of the peptides is shown in Figure 3b. Activity was again higher in bacteria transformed with MCA plasmid (control in grey) than with control vector (control in white), as previously shown in Figure 2a. As observed in promastigote extracts (Figure 3a), peptide 3 decreased trypsin-like activity of MCA expressing bacteria (Figure 3b). Peptides 10 and 11 showed no significant increase in activity, and peptide 1 decreased activity in bacteria, differently from promastigote data. Peptides had no effect on trypsin-like activity of bacteria transformed with control vector.

### Peptides bind to recombinant MCA and to fixed and live parasites

**Figure 4:**
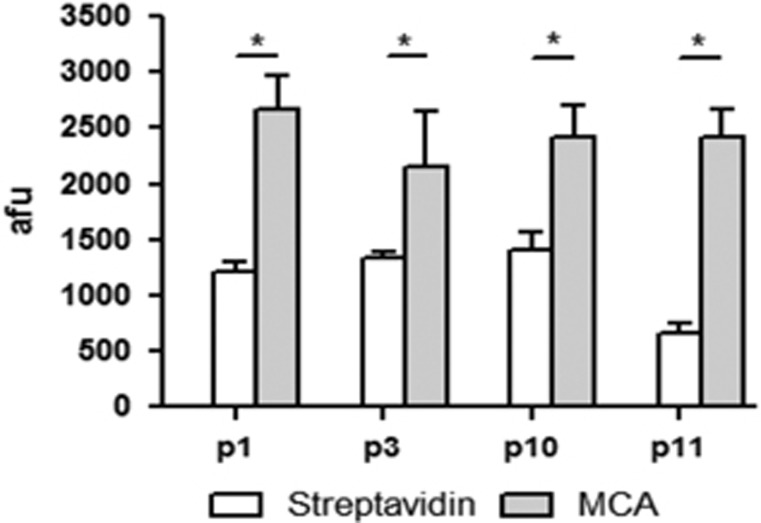
Peptides 1, 3, 10 and 11 bind to MCA. Binding of the four Alexa 488-conjugated peptides to MCA or streptavidin. 15 *μ*g of MCA or streptavidin were immobilized in plates and incubated with 100 *μ*M of each fluorescent peptide. Results of three experiments with technical triplicates. Statistical analysis by ANOVA followed by Tukey. *P<0.05 (MCA *versus* streptavidin binding)

To confirm that peptides modulated activity due to direct interaction with the enzyme we evaluated the binding of peptides 1, 3, 10 and 11 to MCA. Peptides were conjugated with Alexa 488 and incubated with either immobilized recombinant MCA or streptavidin. The results shown in Figure 4 indicate that all peptides have significant higher binding to MCA than to streptavidin, indicating that the phage selection was effective and specific.

**Figure 5:**
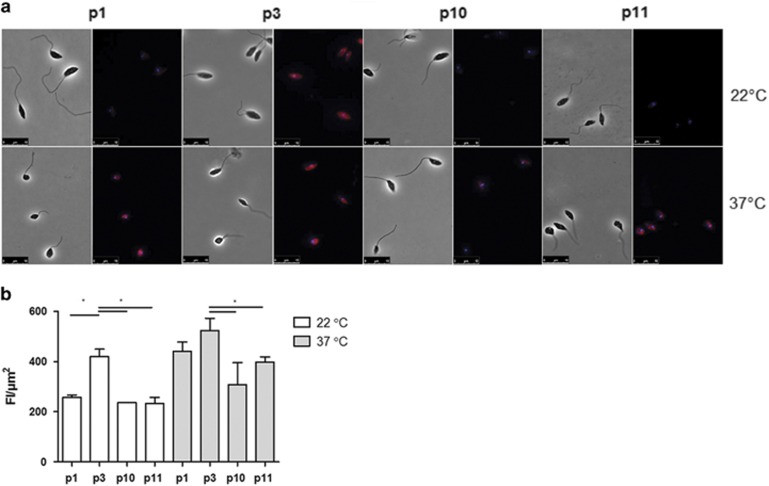
Peptides 1, 3, 10 and 11 bind to fixed parasites previously submitted to heat shock at 37 °C for 1 h. (**a**) Promastigotes were submitted or not to heat shock, immobilized, fixed in glass slides and incubated with Alexa 546 fluorescent peptides 1, 3, 10 and 11, labeled in red. Nuclei and kinetoplasts are labeled with DAPI, in blue. Images of one experiment representative of three with similar profiles. Bars correspond to 10 *μ*m. (**b**) Graph showing quantification of peptide binding to parasites shown in (**a**). Data represent mean fluorescence/*μ*m2 of three promastigotes, determined using ImageJ

We then tested if the peptides were able to recognize and bind to MCA in parasites. To answer this question, we incubated fixed non-permeabilized promastigotes or fixed promastigotes after heat shock (1 h at 37 °C) with peptides conjugated with Alexa 546 and analyzed labeling under the microscope. Representative images shown in Figure 5a and the corresponding quantifications shown in Figure 5b indicate that peptide 3 binds to fixed parasites previously submitted or not to heat shock, while peptides 1, 10 and 11 bind weakly in the absence of heat shock and more intensely to promastigotes submitted to incubation at 37 °C for 1 h. The lower binding of the other peptides compared with peptide 3 suggests that its stronger binding is not artefactual.

### Peptide 3, similar to ecotin, reduces trypsin-like activity and parasite death after heat shock

To test the effect of peptides in live parasites, we investigated whether the four peptides could bind (possibly enter) intact parasites by flow cytometry. Parasites showed labeling with all peptides, with low median fluorescence intensities (MFIs) (Supplementary Figure 2), suggesting that few parasites bound or internalized peptides, probably in low amounts. These results prompted us to evaluate the effect of peptides in live parasites, in an attempt to shed light on the role of the natural MCA ligand.

**Figure 6:**
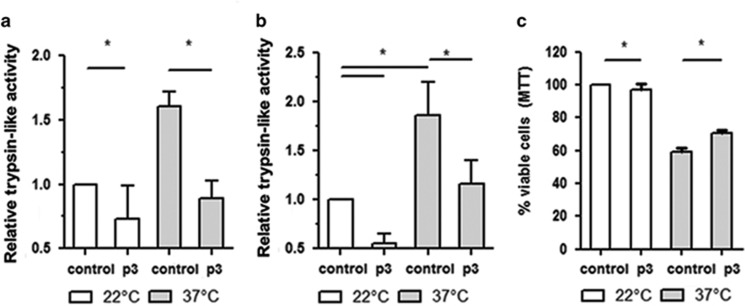
Incubation of parasites with peptide 3 reduces trypsin-like activity and parasite death after heat shock. (**a** and **b**) Relative trypsin-like activity of promastigotes pre-incubated with peptide 3 (100 *μ*M) or DMSO (control), submitted (37 °C) or not (22 °C) to heat shock for 1 (**a**) and 2 (**b**) hours, relative to control at 22 °C. (**c**). Viability (by MTT) of promastigotes pre-incubated with peptide 3 (100 *μ*M) or DMSO (control) submitted (37 °C) or not (22 °C) to heat shock for 2 h, relative to control at 22 °C. Results of three experiments with technical triplicates. ANOVA followed by Tukey, *P<0.05

Considering that peptide 3 binds to immobilized MCA (Figure 4), binds to fixed and live parasites (Figure 5 and Supplementary Figure 2), and alters enzyme activity (Figure 3), we analyzed whether it could modulate trypsin-like activity and parasite death induced by heat shock. Results shown in Figure 6 indicate that peptide 3 significantly reduces parasite trypsin-like activity (a) and parasite death (b) after heat shock. These effects are observed with at least 100 *μ*M of peptide 3 and do not increase with higher peptide concentrations (Supplementary Figure 3).

**Figure 7:**
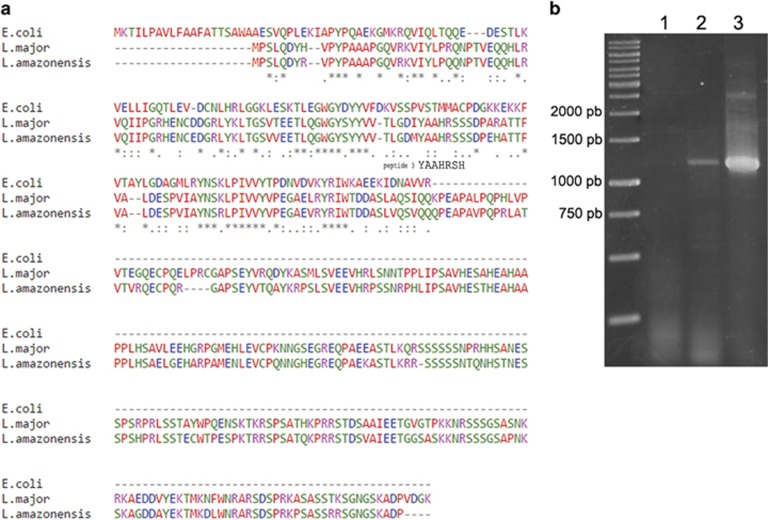
ISP3 sequence and expression in *L. (L.) amazonensis*. (**a**) Alignment of protein sequences of *E. coli* ecotin, *L. major* ISP3 and *L. (L.) amazonensis* (deduced) ISP3, showing peptide 3 corresponding sequence. (**b**) Expression of ISP3 in *L. (L.) amazonensis* promastigotes by RT-PCR. Lane 1: PCR negative control (RNA), lane 2: PCR of cDNA, lane 3: PCR of genomic DNA

A search for possible candidates for peptide 3 indicated high identity (six of the seven amino acids of the peptide) to *L. (L.)* major ISP3, a homologous of bacterial ecotin showing 37% identity to the prokaryotic protein. This protein has not been described nor annotated in *L. (L.) amazonensis*, but the corresponding gene sequence (TriTrypDB) is conserved in this species and the translated protein is 81% identical to *L. major* ISP3, suggesting that peptide 3 corresponds to endogenous *L. (L.) amazonensis* ISP3. Sequence alignment of *L. (L.) amazonensis* ISP3 with *L. major* ISP3 and *Escherichia coli* ecotin is shown in Figure 7a.

RT-PCR using *L. (L.) amazonensis* promastigoteś RNA (Figure 7b) shows that *ISP3* gene is transcribed in this species, indicating that the gene is active and is not a pseudogene.

## Discussion

### *L. (L.) amazonensis* trypsin-like activity correlates with heat shock and promastigote death

We have shown for the first time that heat shock at 37 °C induces *L. (L.) amazonensis* death and increases trypsin-like activity. Previous studies have shown that *Leishmania* MCA increases parasite death after oxidative stress.^20, 21, 25^ Besides, heat shock, oxidative stress and drugs have been shown to alter MCA processing in several *Leishmania* species, including *L. (L.) amazonensis*.^20, 21, 38^

### MCA ligands alter enzyme activity

Regulators of MCA that increase enzyme activity can represent potential drugs for a specific leishmaniasis treatment. We searched for MCA modulators among ligands of the recombinant protein identified using phage display over the recombinant *L. (L.) amazonensis* enzyme. The effects of the peptides on trypsin-like activity were analyzed in *Leishmania* (native enzyme condition) and MCA expressing bacteria extracts, because the purified recombinant enzyme showed very low activity (data not shown). Indeed, MCAs from *L. major*^20, 21^ and probably *L. (L.) amazonensis*, as well as yeast YCA1 (ref. 22) and *Arabidopsis* MCA^23, 24^ require processing to be active, what hampers the purification of an active recombinant enzyme. In fact, the only active *Leishmania* recombinant enzyme described until date was the catalytic domain (equivalent to processed enzyme) of *L. (L.) major* (Gonzalez *et al.*^20^). Five peptides modulated trypsin-like activity in parasite extracts, but only two significantly affected trypsin-like activity in bacteria expressing MCA, both decreasing enzyme activity. Since none of the peptides affected the activity in bacteria not expressing MCA, we concluded that the effects observed were specific for MCA. Promastigotes have enzymes other than MCA with trypsin-like activity, such as cysteine proteinase C,^38^ and have higher activity than MCA expressing bacteria (data not shown). These facts may explain the differences between the results.

The ability of the peptides to bind to MCA was confirmed using recombinant enzyme and fixed parasites submitted or not to heat shock. Heat shock is known to promote enzyme processing, and could also alter enzyme structure and affinity to the peptides, and even open pores in the parasite membrane, facilitating peptide binding. In fact, all peptides showed visible labeling to parasites after heat shock, while peptide 3 was the only peptide with visible binding to parasites at 22 °C, suggesting a more effective interaction with the ‘native' MCA inside the parasite.

### Peptide 3 inhibits MCA and reduces parasite death

The four peptides could represent natural regulators of MCA or could be artificial synthetic mimotopes. To perform functional assays on the effect of the peptides in parasite death, we first checked their ability to bind/enter live cells. Although none of them had the extremely basic amino acid composition or amphipatic membranotropic composition usually found in cell penetrating peptides (CPPs),^39, 40^ peptides 1 and 3 are predominantly basic (three out of seven amino acids) and peptide 11 predominantly nonpolar/hydrophobic (three out of seven amino acids). Peptide labeling was observed in a small proportion of the parasites, suggesting that they bound/entered cells and that functional tests in live parasites could be done.

Functional assays were performed with peptide 3, which significantly reduced trypsin-like activity and parasite death after heat shock. This data indicates that even a small inhibition of MCA by the peptide has an impact on parasite death induced by stimuli such as heat shock. We believe similar effects would be observed if parasite death was triggered by stimuli such as oxidative stress and drugs.

Although peptide 3 decreases cell death and thus could not be used as drug for parasite killing, it may shed light on natural regulators of *Leishmania* MCA. In fact, its similarity to parasite ecotin, more specifically ISP3, suggests that this protein regulates endogenous MCA activity and thus parasite death. First described in *E. coli*, ecotin is a potent inhibitor of several serine proteases.^34^ *L. (L.) major* was shown to have three ecotin-like genes, named ISP 1, 2, 3 for inhibitor of serine peptidase, and ISPs 2 and 3 seem to have a role on the interaction with the host cell.^33^ ISP3 seems to have a very low expression in *L. (L.) major*,^33^ but we were able to identify its transcript in *L. (L.) amazonensis* promastigotes. An ISP was recently identified in *L. (L.) donovani*, and shown to inhibit trypsin activity but not the activity of a *L. (L.) donovani* serine protease.^41^ Ecotin and ISPs are considered inhibitors of serine peptidase from S1A family. Genomic studies have proven that *L. (L.) major* has several serine peptidases belonging to six families but not to S1A family.^42^ Our findings suggest that ecotin may also inhibit cysteine proteases such as MCA. Besides, we show that phage display is an efficient tool to search for ligands and regulators of proteins and enzymes with poorly known pathways.

## Conclusion

Our data demonstrate that *L. (L.) amazonensis* trypsin-like activity and promastigote death are induced by heat shock. We showed that peptides that bind to recombinant MCA, selected by phage display, may affect enzyme activity. One of the peptides, similar to *Leishmania* ISP3, is able to reduce parasite trypsin-like activity induced by heat shock and promastigote death after the shock. We suggest for the first time that ISP3, considered a serine peptidase inhibitor, may also inhibit cysteine proteases such as MCAs.

## Material and methods

### *Leishmania (L.) amazonensis* promastigotes

Promastigotes of *Leishmania (L.) amazonensis* LV79 (MPRO/BR/72/M1841) or M2269 (MHOM/BR/1973/M2269) strains were cultured at 24 °C in M199 medium supplemented with 10% fetal calf serum (FCS). Parasites were sub-cultured every 7 days at inoculums of 2 × 10^6^/ml.

### Heat shock

Promastigotes at day 3 (log phase) were resuspeded at the density of 5 × 10^7^parasites/ml in 116 mM NaCl, 10 mM CaCl2, 5,4 mM KCl, 0,8 mM MgSO_4_, 5,5 mM d-glicose, 50 mM MOPS (3-N-Morpholino propanesulfonic acid pH 7,4) and incubated at 22 or 37 °C in media only or with peptides or DMSO during different periods.

### MTT assay

100 *μ*l of parasites incubated at 22 or 37 °C were transferred to 96 well plates. 20 *μ*l of MTT (MTT (3-[4,5-dimethylthiazol-2-yl]-2,5-diphenyltetrazolium bromide 5 mg/ml in PBS) were added and the plate was incubated at 22 °C for 50 min. 100 *μ*l of SDS 10% were added and absorbance at 595 nm (reference at 655 nm) was measured in a BioTek ELx800 equipment (Biotek., Winooski, VT, USA).

### Annexin V and PI labeling by flow cytometry

Promastigotes at log phase (day 3) were centrifuged at 4000 × *g* for 5 min and washed three times in Hepes buffer (100 mM Hepes, 150 mM NaCl, 5 mM KCl, 3 mM CaCl_2_, 1 mM MgCl_2_, pH 7,2). Parasites were resuspended at 5 × 10^6^ cells/ml in the same buffer and 200 *μ*l were incubated with annexin V Alexa Fluor 488 1 : 200 for 20 min on ice, then washed three times and incubated with 10 *μ*g/ml propidium iodide (IP) for 20 min. As positive control we treated parasites with 100 *μ*M digitonin in annexin V reaction. 30.000 events were captured for each sample in Guava easycyte (Millipore, Bedford, MA, USA).

### Trypsin-like activity of soluble extracts of promastigotes and bacteria

10^8^ promastigotes were lysed in 200 *μ*l of lysis buffer containing 20 mM PIPES, 100 mM NaCl, 1 mM EDTA, 0,1% CHAPS, 10% sucrose, 0,1% Triton X-100 pH 7,2, with 1 mM PMSF, 2 *μ*M Pepstatin A and 50 *μ*M digitonin on ice for 30 min. The lysate was centrifuged at 16 000 × *g* at 4 °C for 5 min, soluble fraction was collected and proteins were measured using Bradford assay (BioRad, SP, Brazil).

10 ml of *E. coli* BL21 (DE3) expressing MCA or containing pET28a plasmid were centrifuged at 16 000 × *g* at 4 °C for 20 min, resuspended in 250 *μ*l lysis buffer containing 0,4 mg/ml lysozyme, and kept on ice for 1 h with vortexing every 10 min. The lysate was then centrifuged as described above.

For both promastigote and bacteria lysates, activity was assayed in 96-well Costar 3603 plates (Costar-Sigma, SP, Brazil) in 100 *μ*l of buffer containing 50 mM Tris-HCl, 15 mM NaCl, 5 mM DTT, 10 mM de CaCl, pH 8.0. Extracts were added in the presence or not of peptides or DMSO and incubated for 2 h at 22 °C. Z-Arg-Arg-AMC substrate was added for 10 *μ*M and capture was performed at 30.5 °C in a POLARstarOmega (BMG, Ortenberg, Germany) fluorimeter excitation at 380 nm and emission at 460 nm.

### SDS-PAGE and western blot

Gels and membranes were prepared as described before,^43^ using 10 *μ*g of proteins and 1 *μ*g of recombinant MCA.

### Production of recombinant metacaspase

Metacaspase (MetaLa) gene was amplified from *L. (L.) amazonensis* using primers based on *L. (L.). mexicana* metacaspase sequence (MetaLaF 5′ATGGCAGACTTTCTTGATATTTTGGGG3′ and MetaLaR 5′TTACCCAGGCGGAGCCG3′). Amplification product was cloned into the pCR4 TOPO sequencing vector (LifeTechnologies, Thermo Fisher, SP, Brazil) and then into the pET28a expression vector, generating pET-28aMeta construct. *Escherichia coli* BL21 (DE3) was transformed with pET-28a and pET-28aMeta, and induced with 0.1 or 1 mM IPTG at 37 °C for 4 h. Bacteria were centrifuged and lysed by sonication (Unique Ultrasonic DES500, SP, Brazil) in 57 mM NaH_2_PO_4_, 1.2 M NaCl pH 7,0 with 0.4 mg/ml lysozyme and 1 mM PMSF. Lysates were centrifuged, filtered in 0.45 *μ*M and transferred to Niquel Ni-NTA column (Qiagen). Column was washed with 57 mM NaH_2_PO_4_ pH 6.0, 128 mM NaCl, 20 mM imidazol and 10% glycerin and recombinant protein was eluted with 500 mM imidazol in wash buffer, dialyzed against 57 mM NaH_2_PO_4_, 1.2 M NaCl pH 7.0 and quantified using Bradford assay (Bio Rad, SP, Brazil).

### Phage display selection

15 *μ*g of MCA 100 *μ*g/ml in 0.1 M NaHCO_3_ pH 8,6 were incubated in 96-well plates (Costar EIA/RIA High binding) at 4 °C o/n. Wells were blocked with 150 *μ*l of 5 mg/ml BSA in 0.1 M NaHCO_3_ pH 8,6 for 1 h at 4 °C, washed six times with TBST (50 mM Tris-HCl pH 7,5, 150 mM NaCl, 0,1% Tween 20) and incubated with 2 × 10^11^ phages from Ph.D.- 7 Phage Display Peptide Library Kit (New England Biolabs, Ipswich, MA, USA) in 100 *μ*l of TBST for 1 h at room temperature. Unbound phages were removed by 10 washing steps in TBST and bound phages were recovered by incubation with 200 *μ*l of exponentially growing (OD_600_=0,5) *E. coli* (ER2738) for 5 min at room temperature. Tittering and amplification were performed as recommended.

### Binding of peptides to metacaspase

15 *μ*g of MCA or 15 ug streptavidin in 150 *μ*l of 0.1 M NaHCO_3_ pH 8,6 were incubated in 96-well plates (Costar3603) at 4 °C o/n. Wells were blocked with 150 *μ*l of 1% BSA in 0.1 M NaHCO_3_ pH 8,6 for 1 h at 4 °C, washed six times with PBS and incubated with 100 *μ*M of Alexa Fluor 488 conjugated peptide (labeled with Protein Labeling Kit- Thermofisher, SP, Brazil) for 1 h at 4 °C. Binding was estimated after analysis in POLARstar Omega at 488 nm for excitation and 520 nm for emission.

### Labeling of parasites with fluorescent peptides

For the binding analysis, promastigotes were washed in PBS, fixed in 4% paraformaldehyde for 30 min, resuspended in PBS and applied in glass slides. After drying, slides were blocked with 1% BSA in PBS for 1 h, washed in PBS and incubated with 100 *μ*M Alexa Fluor 546 peptide in PBS 1% BSA o/n. Slides were then washed in PBS, incubated with 1 *μ*M DAPI in PBS 1% BSA for 1 h, dried and mounted in ProLong (Molecular Probes,Thermo Fisher, SP, Brazil). Images were captured in ZEISS Axio Imager M2 Imaging System (Oberkochen, Germany) and quantification was performed using ImageJ (Bethesda, MD, USA), and expressed as of fluorescence intensity/*μ*m2 (mean values of three parasites normalized by parasite area).

For the evaluation of peptide entry in intact promastigotes we used flow cytometry and the same Alexa Fluor 546 peptides. Promastigotes were incubated for 2 h at 22 °C with 100 *μ*M Alexa Fluor 546 peptide in HEPES buffer 10 mM, washed in PBS and analyzed in Guava easycyte cytometer (Millipore).

### RT-PCR

RNA was isolated from 5 × 10^7^ promastigotes using Trizol reagent (Life Technologies). cDNA was prepared from 2 *μ*g of RNA using random primers, oligodT and Superscript II Reverse Transcriptase (Life Technologies) in 20 *μ*l. PCR was performed with 2 *μ*l cDNA reaction 1 : 10, the corresponding RNA mass or 70 ng of genomic DNA, using 0.7 U Taq polymerase (Life Technologies) and the following cycle conditions: 94 °C for 4 min, 35 cycles of 94 °C 30 s, 60 °C 45 s, 72 °C 2 min and 72 °C for 10 min.

### Statistical analysis

GraphPad software (San Diego, CA, USA) was used to perform all analysis. We employed one way ANOVA followed by Tukey's multiple comparison test (for three or more samples), or *t*-test (for comparison of two conditions).

## Figures and Tables

**Table 1:** Peptide sequences and frequencies of the 50 phages




Peptide numbers (column 1), peptide sequences showing polar amino acids in green, hydrophobic in red, acid in blue and basic in pink (column 2), frequencies of the sequences (column 3)
