# Supplementary material for: Metacaspase-binding peptide inhibits heat shock-induced death in Leishmania (L.) amazonensis
Source: Cell Death Dis. 2017 Mar 2;8(3):e2645–. doi: 10.1038/cddis.2017.59 (PMC5386556; doi:10.1038/cddis.2017.59)
Supplement: Supplementary Table I [file cddis201759x1.docx]

| **Input** | **Output** | | |
| --- | --- | --- | --- |
|  | Cycle 1 | Cycle 2 | Cycle 3 |
| 2x 10^11^ pfu | 17,8 x 10^5^ pfu | 20 x 10^5^ pfu | 26 x 10^5^ pfu |
