# Supplementary figures and images for: Metacaspase-binding peptide inhibits heat shock-induced death in Leishmania (L.) amazonensis
Source: Cell Death Dis. 2017 Mar 2;8(3):e2645–. doi: 10.1038/cddis.2017.59 (PMC5386556; doi:10.1038/cddis.2017.59)

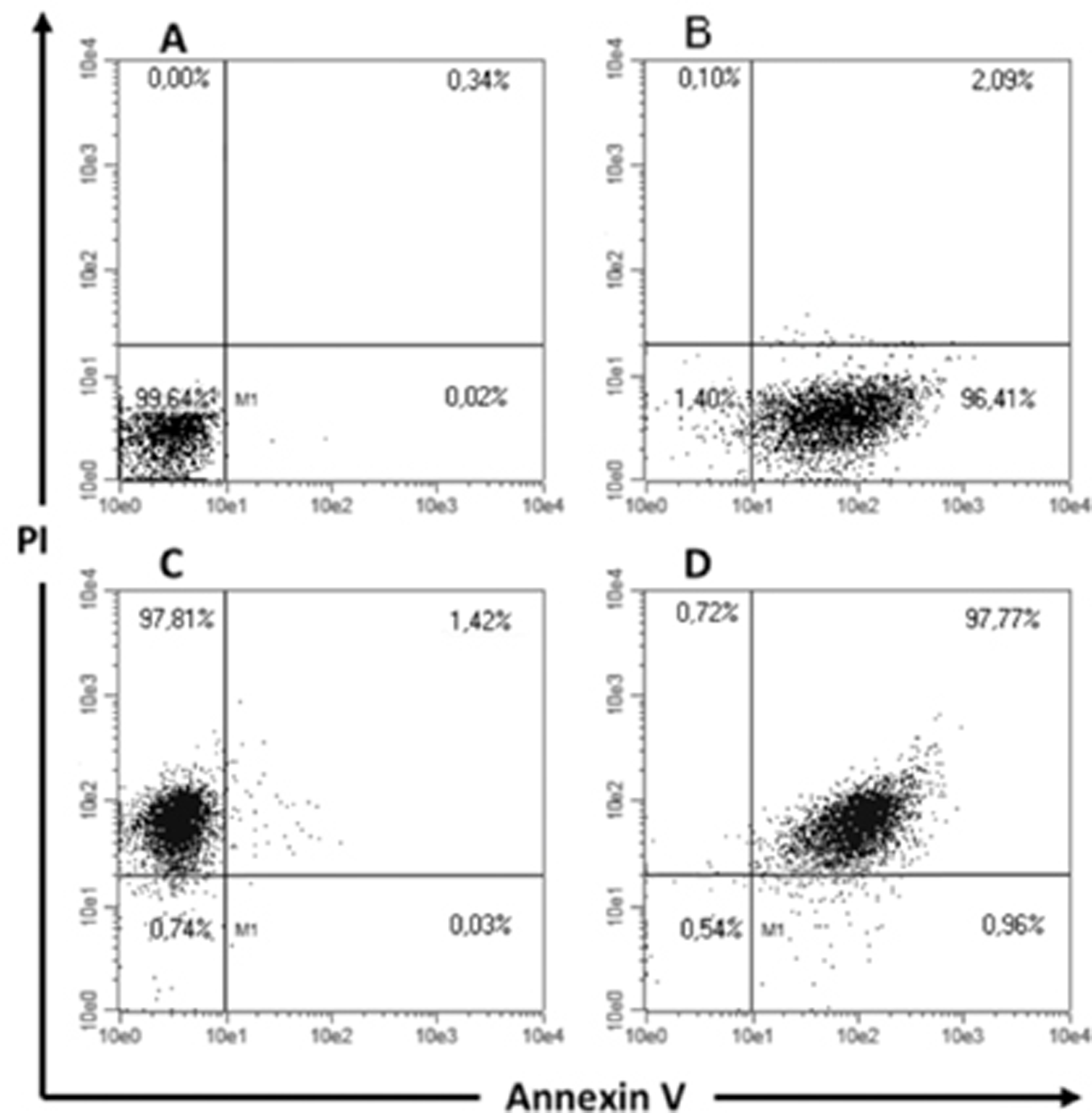

Supplement: Supplementary Figure 1 [file cddis201759x2.tif]

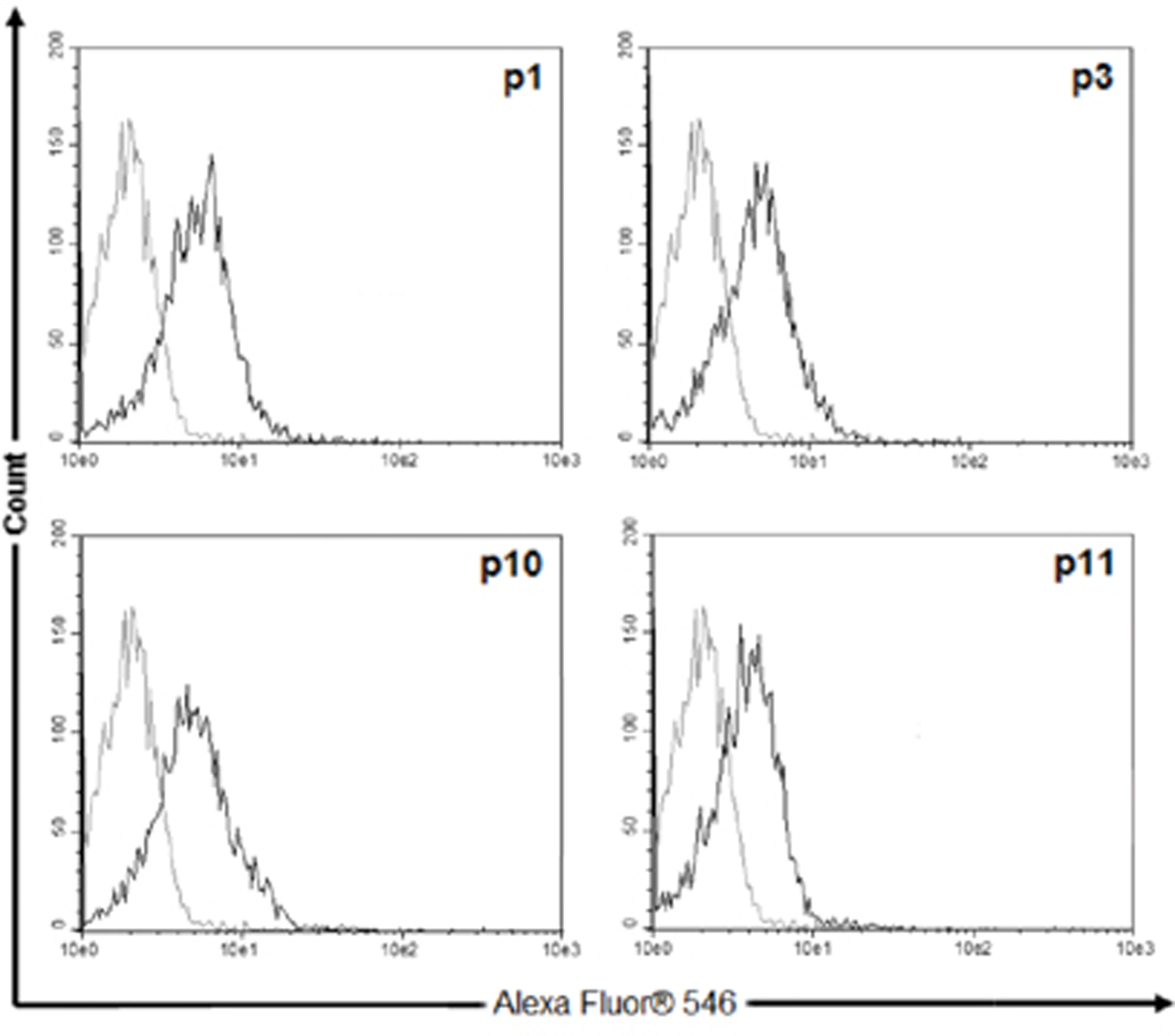

Supplement: Supplementary Figure 2 [file cddis201759x3.tif]

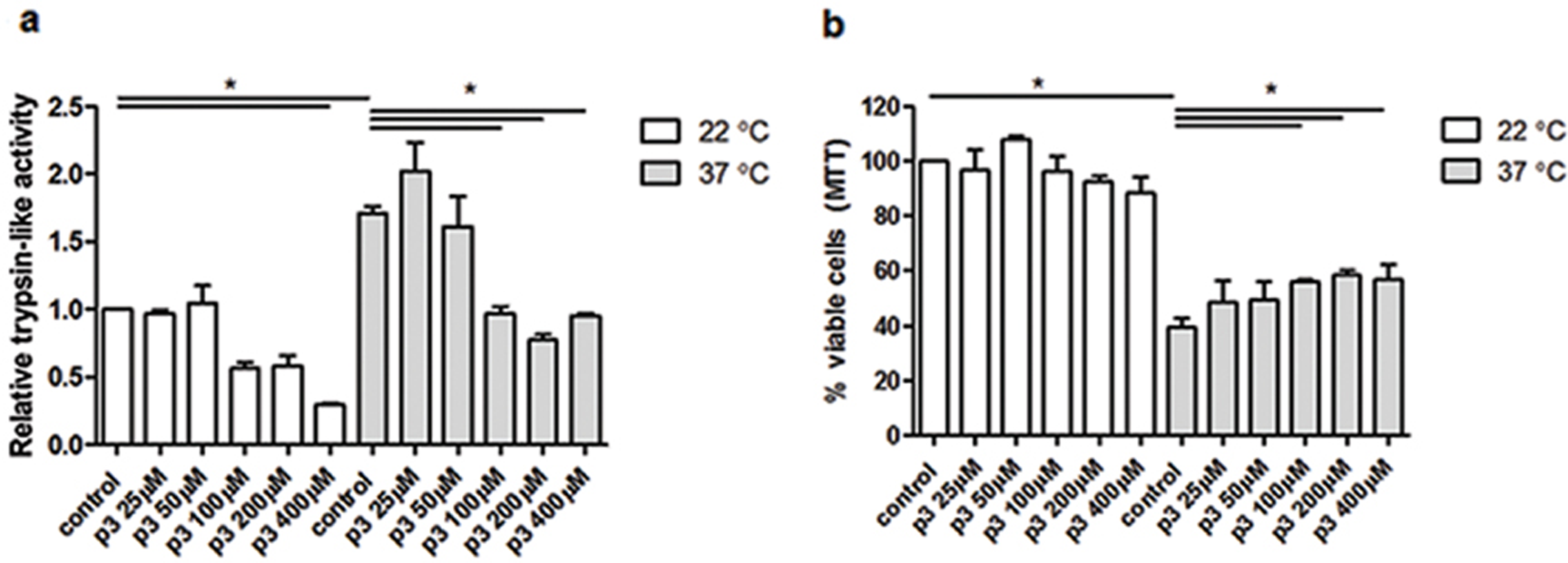

Supplement: Supplementary Figure 3 [file cddis201759x4.tif]
